# YY1-induced DLEU1/miR-149-5p Promotes Malignant Biological Behavior of Cholangiocarcinoma through Upregulating YAP1/TEAD2/SOX2

**DOI:** 10.7150/ijbs.66224

**Published:** 2022-07-04

**Authors:** Jinglin Li, Xingming Jiang, Yi Xu, Pengcheng Kang, Peng Huang, Nanfeng Meng, Hang Wang, Wangyang Zheng, Hao Wang, Zhidong Wang, Xiangyu Zhong, Yunfu Cui

**Affiliations:** Department of General Surgery, The 2nd Affiliated Hospital of Harbin Medical University, 148 Baojian Street, Harbin 150086, Heilongjiang Province, China.

**Keywords:** cholangiocarcinoma, prognosis, DLEU1, miR-149-5p, YAP1

## Abstract

Cholangiocarcinoma is an extremely malignant cancer with poor prognosis. Finding efficient diagnosis and treatment is the indispensable way to improve the prognosis of CCA patients. Therefore, exploring molecular abnormalities in CCA development is urgently needed. DLEU1 is a potential tumor-related lncRNA and abnormally expressed in multiple cancers. In this study, TCGA data analysis showed upregulation of DLEU1 expression in CCA. Furthermore, we confirmed that DLEU1 expression was increased in CCA tissues and cells compared with corresponding controls. Upregulated DLEU1 was related to poor clinicopathological characteristics. Functionally, silencing DLEU1 inhibited CCA proliferation, invasion, stemness maintenance and chemo-resistance, whereas amplifying DLEU1 promoted malignant biological behavior of CCA cells. Mechanistically, DLEU1 expression was transcriptionally facilitated by transcription factor YY1. Moreover, DLEU1 promoted oncogene YAP1 expression by functioning as a sponge to competitively bind to miR-149-5p. YAP1 promoted CCA proliferation, invasion and stemness maintenance, whereas miR-149-5p inhibited malignant biological behavior of CCA. Rescue experiments confirmed that the cancer-promoting effect of DLEU1 was saved by interfering miR-149-5p or YAP1. Furthermore, YAP1 promoted tumor stemness maintenance partly by acting as a transcriptional coactivator to promote TEAD2-induced SOX2 expression. These findings indicated that YY1-induced DLEU1 played a crucial role in CCA progression via miR-149-5p/YAP1/TEAD2/SOX2 axis.

## Introduction

Cholangiocarcinoma (CCA) is a malignant cancer of digestive system caused by malignant transformation of bile duct epithelial cells or the transdifferentiation from mature hepatocytes to malignant cholangiocytes 1-3. Complex anatomy and single treatment are the main causes of poor prognosis in patients with CCA. Furthermore, most patients have reached advanced stage of the tumor due to lack of sensitive early diagnostic markers. As a result, these patients lost their best chance of surgery. At present, the treatment of CCA with radiotherapy and chemotherapy cannot achieve satisfactory outcome 4. Sensitive biomarkers and effective therapeutic targets are pivotal points to improve CCA prognosis. To explore the molecular mechanism of CCA development and to find the key pathways leading to the canceration are urgently needed.

Thanks to rapid progress in genome sequencing technology, a large number of non-coding RNAs once considered to be transcription by-products have attracted widely attention, such as long noncoding RNA (lncRNA), microRNA (miRNA), circular RNA (circRNA) 5. LncRNAs represent a group of non-protein-coding RNA with longer than 200 nt in size. The RNAs possess limited protein coding capability by reason of lacking evident open reading frame 6. LncRNAs have been identified as key players in gene expression regulation. They can be involved in disease progression through multiple mechanisms at epigenetic, transcriptional and post-transcriptional levels, such as competitive endogenous RNA (ceRNA), protein scaffold, signal, decoy, transcript guide 7-9. Accumulating evidence suggested that lncRNAs functioned as cancer suppressors or oncogenes in malignant biological progression of tumors 10. Deleted in lymphocytic leukemia 1 (DLEU1) is a type of lncRNA and maps to chromosome 13q14.3. DLEU1 has been shown to exhibit crucial carcinogenic roles in multiple tumors, including hepatocellular carcinoma, pancreatic ductal adenocarcinoma, colorectal cancer, and gastric cancer 11-14. For example, upregulated DLEU1 was observably correlated with neural invasion and poor differentiation in pancreatic ductal adenocarcinoma 12; DLEU1 overexpression promoted tumor cell invasion and growth in colorectal cancer 13. Nevertheless, the role of DLEU1 in cholangiocarcinoma progression remains obscure. The miRNAs are another type of non-coding RNAs and also participate in the development of various diseases. They can bind to other sequences like lncRNA and mRNA through complementary binding sites. Thus miRNAs usually regulate gene expression by ceRNA method at the post-transcriptional level 15. MiR-149-5p is a tumor suppressor gene in diverse tumor pathological processes. For instance, miR-149-5p inhibited cell growth and predicted favorable survival in human osteosarcoma 16.

Yes-associated protein 1 (YAP1) functions as a tumor-promoting gene in multiple cancers, and ectopic expression of YAP1 promotes oncogenic transformation 17. YAP1 is a transcription coactivator by reason of lacking DNA-binding ability. Thus YAP1 has to physically interact with DNA-binding transcription factors, and then transcriptionally activate downstream genes 18. YAP1 binds to DNA-binding transcription factors by its N-terminal region. Transcriptional enhanced associated domain (TEAD) transcription factors are the main binding partner for YAP1, and they work together to exert cancer-promoting function 19. Four types of TEADs (TEAD1, TEAD2, TEAD3, TEAD4) are generally expressed in human organs with variant tissue distribution 20.

Cancer stem cells (CSCs) represent a subgroup of tumor cells with potential driving forces of tumor initiation and progression. They possess peculiar functionalities such as self-renewal, pluripotency, plasticity and multilineage differentiation 21. Thus they play crucial roles in initiation, metastasis, relapse and chemo-resistance of tumors. CSCs have become an important target for cancer therapy. Tumor microenvironment and signaling pathways involved in CSCs function are complex and diverse. Nanog, octamer-binding protein 4 (OCT4), SRY-box 2 (SOX2) and Kruppel-like factor 4 (KLF4) are key participants of tumor stemness 22. In-depth studies of these molecules and their upstream and downstream pathways contribute to the treatment of tumors.

The present research first demonstrated that DLEU1 was significantly increased and related to awful prognosis in cholangiocarcinoma. Yin Yang 1 (YY1)-mediated DLEU1 boosted tumor growth, metastasis, epithelial-mesenchymal transition (EMT), stemness maintenance and drug resistance *in vitro* and* in vivo*. DLEU1 competitively bound to miR-149-5p to upregulate oncogene YAP1, and then YAP1 bound to TEAD2 to transcriptionally activate SOX2. Taken together, DLEU1 acts as a cancer-promoting gene and exhibits pivotal function in CCA progression.

## Materials and methods

### The Cancer Genome Atlas (TCGA) dataset analysis

The gene expression profile of CCA was obtained from TCGA data portal (https://tcga.xenahubs.net). Differential expression analysis was conducted with the package limma of R statistical software. The false discovery rate < 0.05 and |log2 (fold change)| > 1 were set as the threshold.

### Clinical tissue and data

A total of 55 pairs of CCA tissues and paired adjacent nontumor bile duct tissues were obtained from The 2^nd^ Affiliated Hospital of Harbin Medical University. After resection, the samples were promptly frozen and preserved in liquid nitrogen. Patients with preoperative chemotherapy and radiotherapy had been excluded.

### Cell culture and transfection

RBE and HCCC-9810 were purchased from the Cell Bank of Chinese Academy of Sciences (Shanghai, China). QBC939, CCLP-1 and HIBEC were stored in our laboratory. The cells were cultured using DMEM and RPMI-1640 supplemented with 10% FBS (Invitrogen, Carlsbad, CA). SiRNA and pcDNA3.1 plasmid (GenePharma, Shanghai, China) were designed and purchased for gene knockdown and overexpression. Lipofectamine 3000 (Invitrogen) was used to transfection according to the manufacturer's directions. The transfection sequences are shown in Table S1.

### qRT-PCR

RNAs was drawn from tissue samples and cell lines according to the protocol of TRIzol reagent (Invitrogen). Sample RNA was reverse transcribed using Transcriptor First Strand cDNA Synthesis Kit (Roche, Penzberg, Germany). Each sample was amplified in a 20 μl reaction mixture using FastStart Universal SYBR Green Master (Roche). We used the 2^-ΔΔCt^ method to convert the fold changes. Relative expression levels were calculated and normalized to endogenous GAPDH or U6. The primer sequences are shown in Table S1.

### Western blot

Total proteins were extracted from tumor tissues and cultured cells by radio immunoprecipitation assay lysis buffer (Beyotime, Beijing, China). The protein lysates were separated by 12% SDS-PAGE and then transferred onto 0.45 μm PVDF membrane (Millipore, Billerica, MA). 5% fat-free milk was used to block nonspecific combination at 37 °C for 2 h. Afterwards, the membranes were incubated by diluent primary and secondary antibodies (Cell Signaling Technology, Danvers, MA). GAPDH was endogenous control.

### Proliferation assays

The cells were measured at 0, 24, 48, 72, and 96 h. Before each test, the supernatant of each group was removed, and then cells were incubated in culture medium with 10 μl/well CCK-8 (Dojindo, Kumamoto, Japan) for 2 h. The optical density value for each well was measured at 450 nm.

Transfected cells were cultured in 96-well plates. 100 μl 5-ethynyl-2'-deoxyuridine (EdU) diluent (Ribobio, Guangzhou, China) was added into the plates, which were incubated for 2 h. After that, the samples were stained with Apollo 567, and cell nuclei were stained with Hoechst 33342. EdU-positive cells were counted under a fluorescence microscope (Leica, Wetzlar, Germany).

The cells were equably seeded into 6‐well plate with complete culture medium for 12 days. Paraformaldehyde was used to fix and crystal violet (Beyotime) was used to observe.

### Metastasis assays

A linear scratch was made using a pipette tip on cellular monolayer surface in wound healing assay. After culturing in serum-free medium for 0 and 48 h, wound closure situation were recorded. Transwell assay was implemented according to standard protocols as previously described (Li et al., 2020).

### Chemo-resistance assay

Gemcitabine (MedChem Express, Monmouth Junction, NJ) or cisplatin (Sigma, St. Louis, MO) was used to treat transfected cells with concentration gradient or time gradient. CCK-8 assay was implemented to detect cytotoxicity of cisplatin and gemcitabine. The concentration of cisplatin and gemcitabine in time gradient assays was 4 μM and 20 μM.

For *in vivo* assay, transfected CCLP-1 cells were used to subcutaneously inject into the posterior flanks of BALB/c nude mice. After 1 week, the mice were intraperitoneally injected with PBS, cisplatin (5 mg/kg) or gemcitabine (50 mg/kg) twice a week for 2 weeks.

### Subcellular fractionation assay

PARIS kit (Life Technologies, Carlsbad, CA) was used to isolate nuclear and cytoplasmic fractions. Cell fractionation buffer was used to incubate the samples. Next, the cell lysates were centrifugated for acquiring upper cytoplasmic components. Nuclear pellet was lysed using cell disruption buffer.

### Tumor xenograft assay

5-week-old female BALB/c nude mice were purchased from Vital River Laboratory Animal Technology Co., Ltd. (Beijing, China). Transfected QBC939 cells were used to subcutaneously inject into posterior flanks of the mice. Tumor volumes were measured per three days (0.5 × length × width^2^). Tumor weights were measured after 21 days.

### Spheroid formation, ChIP, RIP and luciferase reporter assays

Spheroid formation, ChIP, RIP and luciferase reporter assays were implemented according to standard protocols as previously described (Li et al., 2020).

### Statistical analysis

The results were shown as mean ± SD from at least 3 independent experiments. Data analyses used GraphPad Prism 6.0 (GraphPad Software, La Jolla, CA) and SPSS 20.0 (IBM SPSS, Armonk, NY). Chi-square test, *t*-test and ANOVA were used to compare the differences between groups. Prognostic risk factors were evaluated by Cox regression model and receiver operating characteristic curve (ROC) analysis. p value < 0.05 was statistically significant.

## Results

### DLEU1 was upregulated and represented poor prognosis in CCA

**Figure 1:**
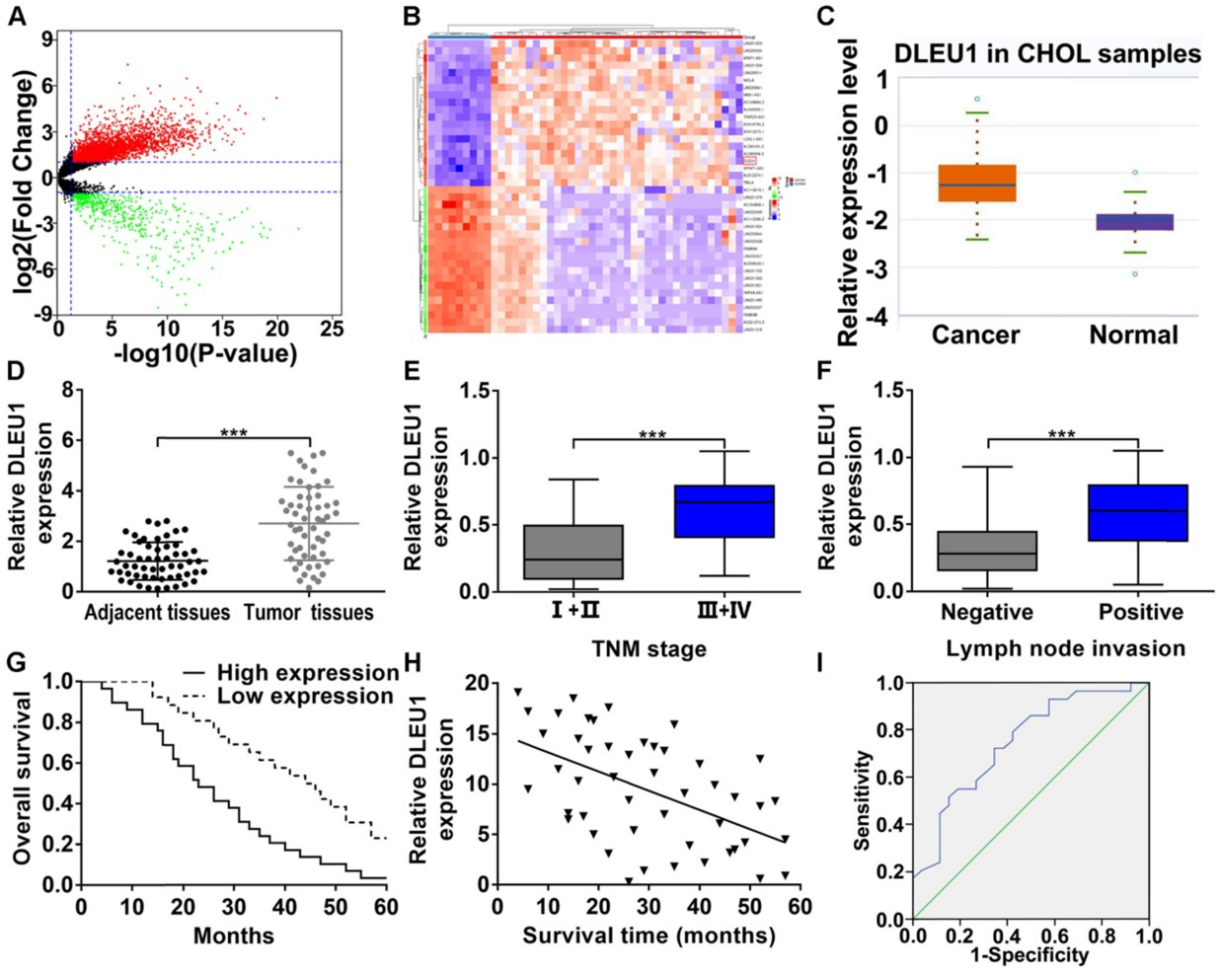
** DLEU1 level and clinical relevance in CCA. (A)** Volcano plot of differentially expressed lncRNAs in CCA from TCGA dataset (*t*-test). (B) Heatmap of the top 20 upregulated and downregulated lncRNAs with significant changes (*t*-test). **(C)** Detection of DLEU1 expression in CCA samples of TCGA database (*t*-test). **(D)** DLEU1 level was upregulated in CCA tissues (*t*-test). **(E)** DLEU1 level was higher at TNM III+IV stage than TNM I+II stage (*t*-test). **(F)** DLEU1 level was higher in patients with lymph node invasion (*t*-test). **(G)** Kaplan-Meier certified that worse OS occurred in patients with high DLEU1 expression (log rank test). **(H)** DLEU1 level and OS of patients were negatively correlated (linear regression). **(I)** ROC analysis was conducted to assess the prognostic correlation of DLEU1. ^***^p < 0.001.

The gene expression profile of CCA was downloaded from TCGA database, and we analyzed the differentially expressed lncRNAs (Figure 1A). The top 20 upregulated and downregulated lncRNAs with significant changes were picked (Figure 1B). Among these picked lncRNAs, DLEU1 has been corroborated to be elevated in multifold tumors (liver cancer, pancreatic ductal adenocarcinoma, colorectal cancer, and gastric cancer). However, its function in CCA is still unclear. TCGA database indicated that DLEU1 was enhanced in CCA (p < 0.001; Figure 1C). Indeed, DLEU1 was rasied in CCA tissues contrasted with control group (Figure 1D). Besides, DLEU1 was linked to advanced TNM stage and lymph node invasion (Figures 1E, F; Table 1). Survival data certified that worse overall survival (OS) occurred in patients with high DLEU1 expression (log rank p < 0.001; Figure 1G). Furthermore, DLEU1 level and OS of patients were certified to be negatively correlated (r = -0.5292, p < 0.001; Figure 1H). Cox regression analysis testified that advanced TNM stage, high DLEU1 level and lymph node invasion were linked to CCA prognosis, and the first two were independent risk indicator of prognosis (Table 2). As a prognostic indicator, the value of area under curve (AUC) of DLEU1 was 0.747 (95% CI: 0.618-0.877) with 65.4% specificity and 72.4% sensitivity (p < 0.001; Figure 1I).

### DLEU1 boosted tumor proliferation, invasion and EMT

**Figure 2:**
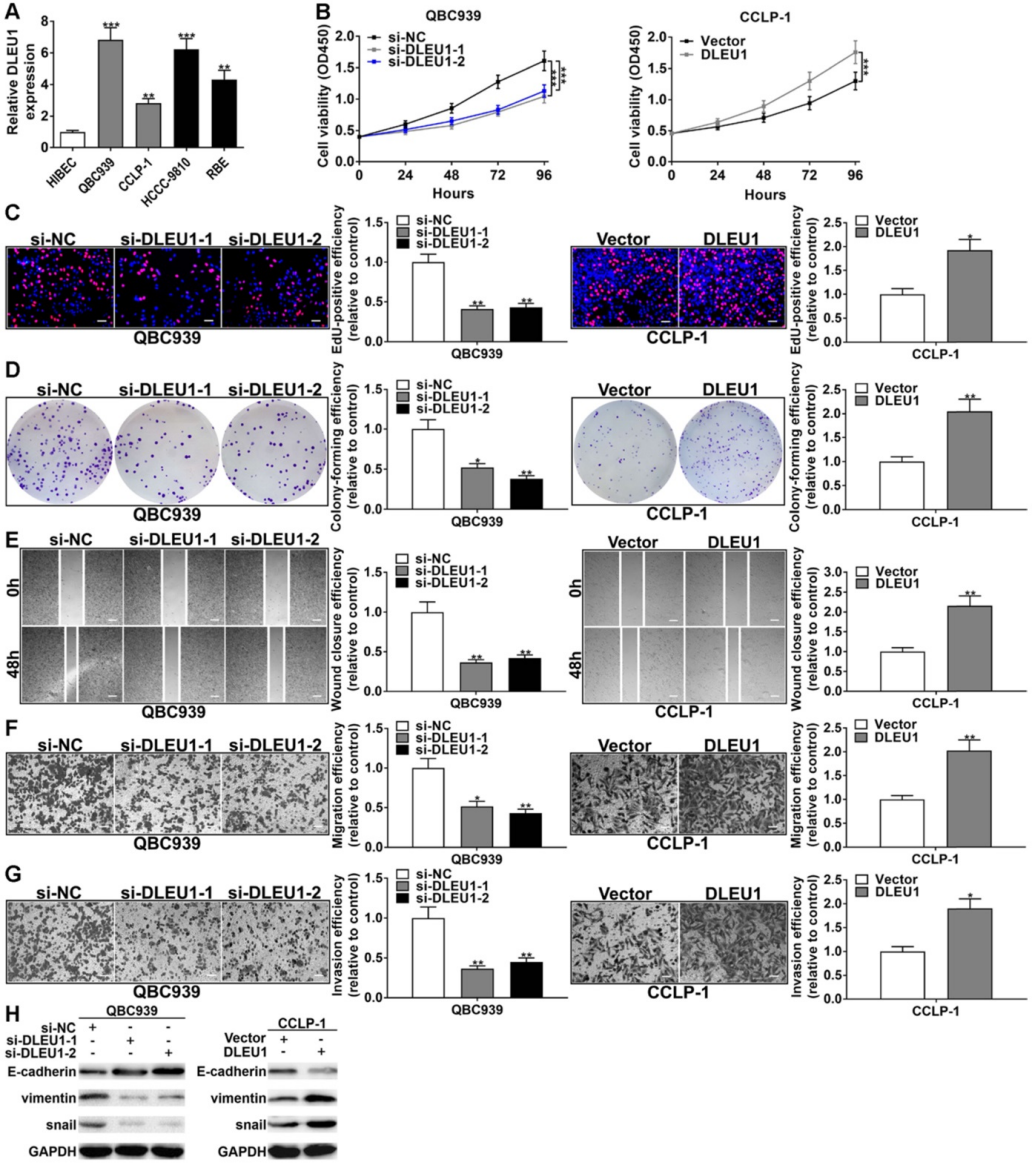
** DLEU1 boosted CCA cell proliferation and invasion. (A)** DLEU1 level in CCA cells and biliary epithelial cells (one-way ANOVA). **(B)** CCK-8 assay (two-way ANOVA) and **(C)** EdU assay corroborated that DLEU1 boosted CCA cell proliferation (*t*-test). (D) DLEU1 boosted colony formation of CCA cells (*t*-test). **(E)** Wound healing assay (*t*-test) and **(F-G)** transwell assay corroborated that DLEU1 boosted CCA cell migration and invasion (*t*-test). (H) DLEU1 boosted tumor EMT process (two-way ANOVA). ^*^p < 0.05, ^**^p < 0.01, ^***^p < 0.001.

DLEU1 was enhanced in CCA cells contrasted with normal HIBEC (Figure 2A). QBC939 and CCLP-1 were determined for further study base on qRT-PCR data. The efficiencies of si-DLEU1 and pcDNA3.1-DLEU1 were satisfactory (Supplementary Figure 1). The si-DLEU1-1 and si-DLEU1-2 were applied due to better knockdown. The proliferative curve of CCK-8 assays showed that QBC939 viability was significantly suppressed in si-DLEU1 cells, but upregulated DLEU1 boosted CCLP-1 proliferation contrasted with controls (Figure 2B). In EdU incorporation assays, silencing DLEU1 depressed tumor cell proliferation, while enhanced DLEU1 boosted cellular proliferation (Figure 2C). Besides, silencing DLEU1 depressed CCA colony formation, whereas enhanced DLEU1 boosted this capability (Figure 2D). In wound healing assay, decreased DLEU1 depressed QBC939 migration but enhanced DLEU1 contributed to CCLP-1 migration (Figure 2E). Transwell data demonstrated that DLEU1 contributed to tumor migration and invasion as well (Figure 2F, G). Furthermore, DLEU1 boosted EMT process by reducing E-cadherin level and augmenting snail and vimentin (Figure 2H).

### DLEU1 boosted CCA stemness maintenance and chemo-resistance

**Figure 3:**
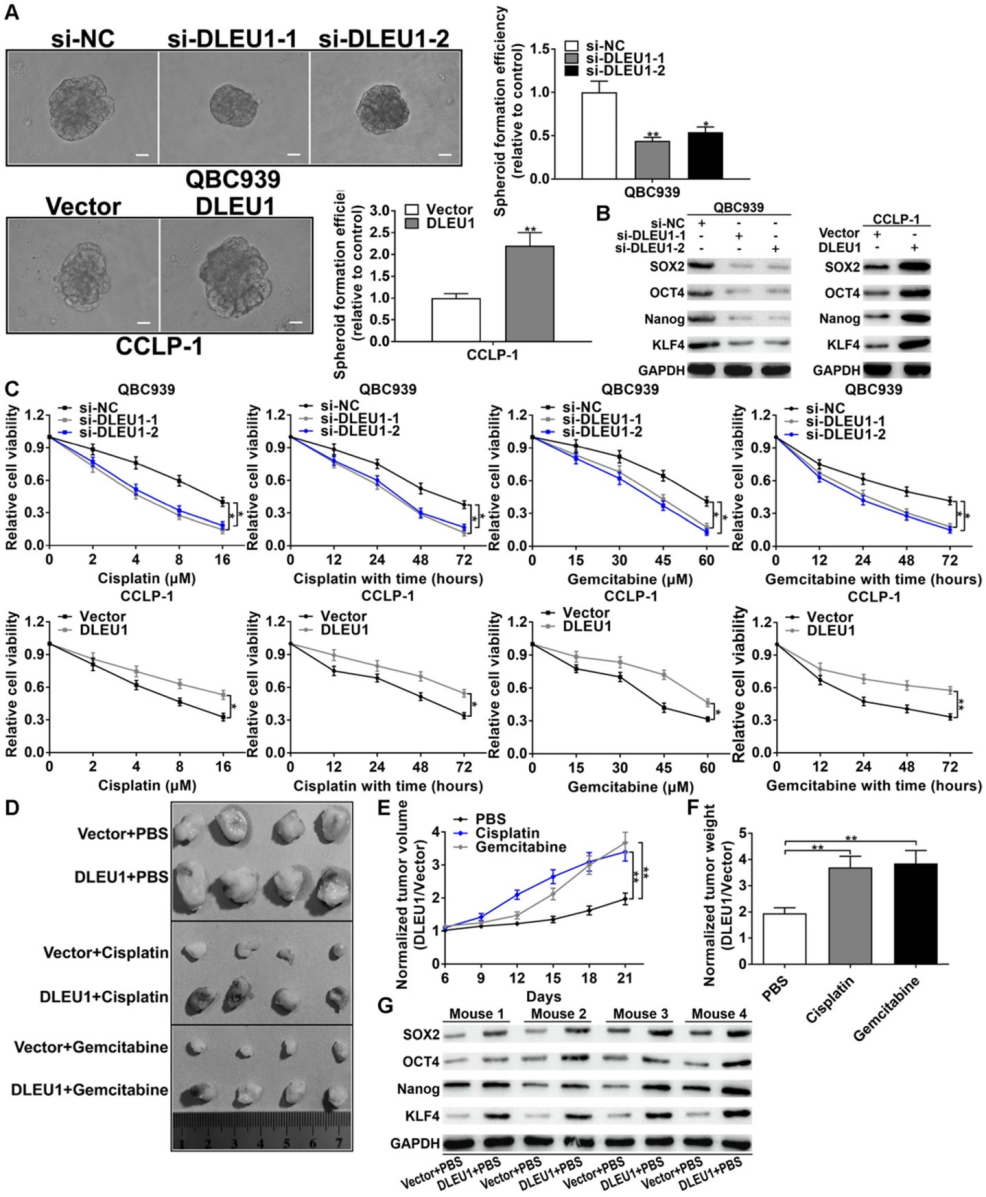
** DLEU1 boosted tumor stemness maintenance and chemo-resistance. (A)** DLEU1 boosted spheroid formation of CCA cells (*t*-test). **(B)** DLEU1 boosted stem cell markers (SOX2, OCT4, Nanog, KLF4) expression (two-way ANOVA). **(C)** DLEU1 boosted chemo-resistance of cisplatin and gemcitabine in CCA cells (two-way ANOVA) and **(D)** in nude mice. **(E-F)** Normalized tumor growth curve (two-way ANOVA) and tumor weight (*t*-test) showed that DLEU1 significantly increased chemo-resistance to cisplatin and gemcitabine treatment *in vivo*. **(G)** DLEU1 boosted the expression of stem cell markers* in vivo* (two-way ANOVA). ^*^p < 0.05, ^**^p < 0.01.

CSCs are the trigger of tumorigenesis and the initiator of tumor drug resistance. In this study, silencing DLEU1 depressed tumor spheroid formation, but enhanced DLEU1 boosted the formation of tumor spheroid (Figure 3A). Afterwards, the stem cell markers (SOX2, OCT4, Nanog, KLF4) were decreased in si-DLEU1 cells but increased in pcDNA3.1-DLEU1 cells (Figure 3B). In addition, cisplatin or gemcitabine was used to treat CCA cells in time-dependent and dose-dependent modes. CCK-8 data demonstrated that DLEU1 augmented resistance of CCA cells to cisplatin and gemcitabine (Figure 3C). For *in vivo* confirmation, nude mice were subcutaneously injected with CCLP-1 transfected with pcDNA3.1-DLEU1. Subsequently, PBS, cisplatin or gemcitabine was used to intraperitoneally inject into mice. The results documented that enhanced DLEU1 boosted tumor growth contrasted with vector controls, while cisplatin or gemcitabine treatment markedly restrained tumor growth compared with PBS controls (Figure 3D, Supplementary Figure 2). Because DLEU1 overexpression promoted tumor growth, which made us difficult to judge whether DLEU1 could enhance chemo-resistance *in vivo* from the absolute tumor volumes and weights. Thus we normalized the tumor volumes and weights to the vector control of each group. The results showed that DLEU1 enhanced the chemo-resistance of tumors including volumes and weights on treatment of cisplatin and gemcitabine *in vivo* (Figures 3E, F). Besides, DLEU1 upregulated stem cell markers (SOX2, OCT4, Nanog, KLF4) in DLEU1 + PBS group contrasted with vector + PBS group (Figure 3G).

### YY1 induced DLEU1 expression at transcriptional level

**Figure 4:**
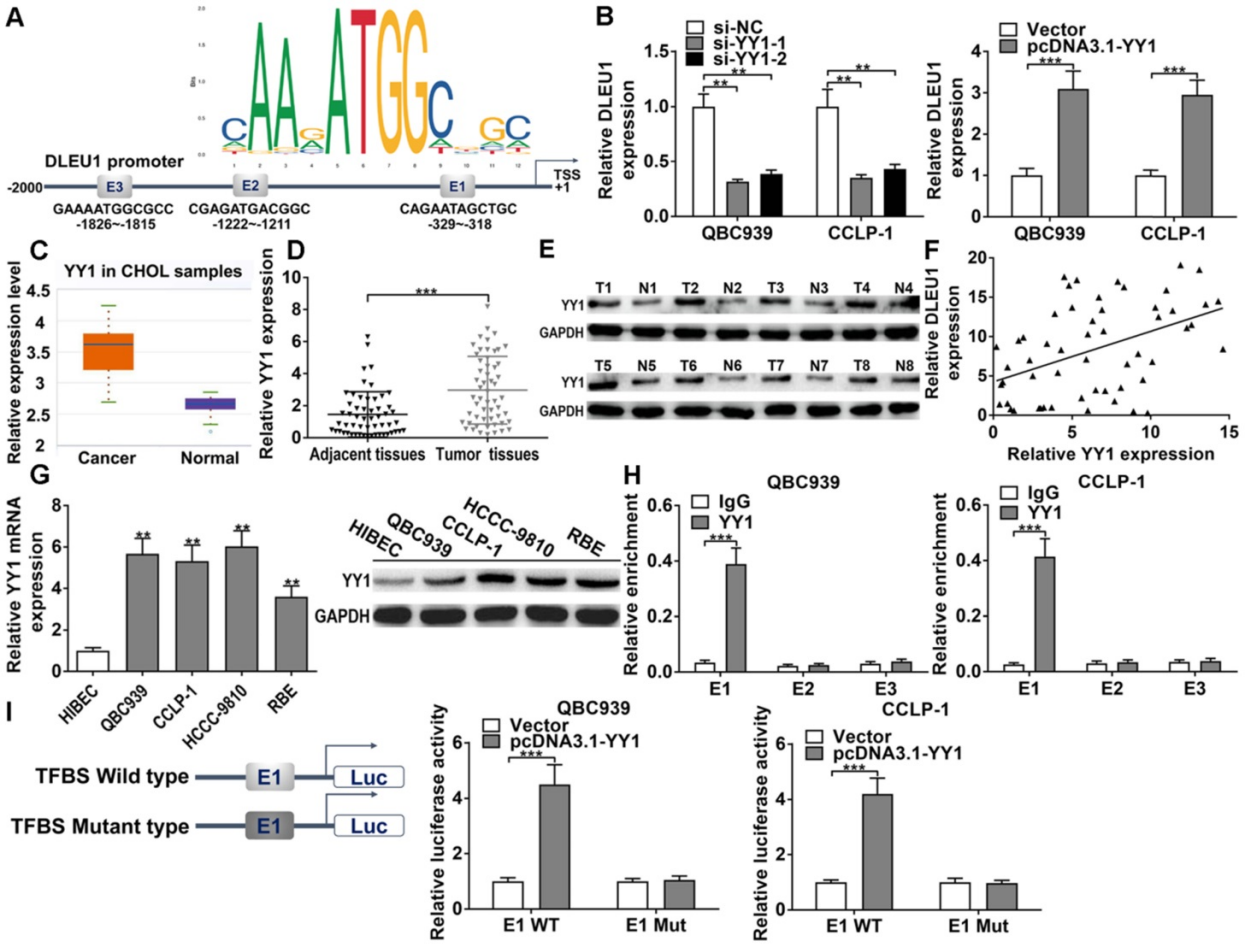
**DLEU1 was mediated via YY1. (A)** YY1 motif and binding sites (E1, E2, E3) to DLEU1 promoter forecasted via JASPAR. **(B)** Decreased YY1 restrained DLEU1 expression and upregulated YY1 facilitated DLEU1 expression (*t*-test). **(C)** Detection of YY1 expression in CCA samples of TCGA database (*t*-test). **(D-E)** YY1 level was upregulated in CCA tissues (*t*-test). **(F)** YY1 level was positively related to DLEU1 level (linear regression). **(G)** YY1 level in CCA cells and biliary epithelial cells (one-way ANOVA). **(H)** ChIP documented that YY1 antibody obviously increased E1 fragments (*t*-test). **(I)** YY1 activated wild type E1 luciferase plasmid rather than mutant E1 plasmid (*t*-test). ^**^p < 0.01, ^***^p < 0.001.

In DLEU1 promoter, we found 3 binding sites of YY1 (E1, E2, E3) via JASPAR database (http://jaspar.genereg.net/) (Figure 4A). The efficiencies of si-YY1 and pcDNA3.1-YY1 in CCA cells were successful (Supplementary Figure 3A). As predicted, YY1 knockdown decreased DLEU1 transcription and YY1 overexpression increased DLEU1 transcription verified by qRT-PCR in QBC939 and CCLP-1 cells (Figure 4B). Moreover, YY1 level was augmented in CCA by TCGA analysis (p < 0.001; Figure 4C). After further detection of our experiments, YY1 mRNA was enhanced in CCA tissues contrasted with controls (Figure 4D), and YY1 protein expression was also increased in CCA tissues confirmed by western blot (Figure 4E). Moreover, YY1 expression was positively related to DLEU1 expression in CCA tissues (r = 0.4482, p < 0.001; Figure 4F). Besides, YY1 expression was increased in tumor cells (Figure 4G). ChIP documented that YY1 antibody obviously increased E1 fragments (Figure 4H). Luciferase plasmids with wild type or mutant type of E1 were constructed. YY1 activated wild type E1 luciferase plasmid rather than mutant E1 plasmid (Figure 4I). The data illustrated that YY1 was an upstream regulator of DLEU1, and induced DLEU1 expression via transcription factor binding site (TFBS) E1.

### DLEU1 competitively bound miR-149-5p in CCA

**Figure 5:**
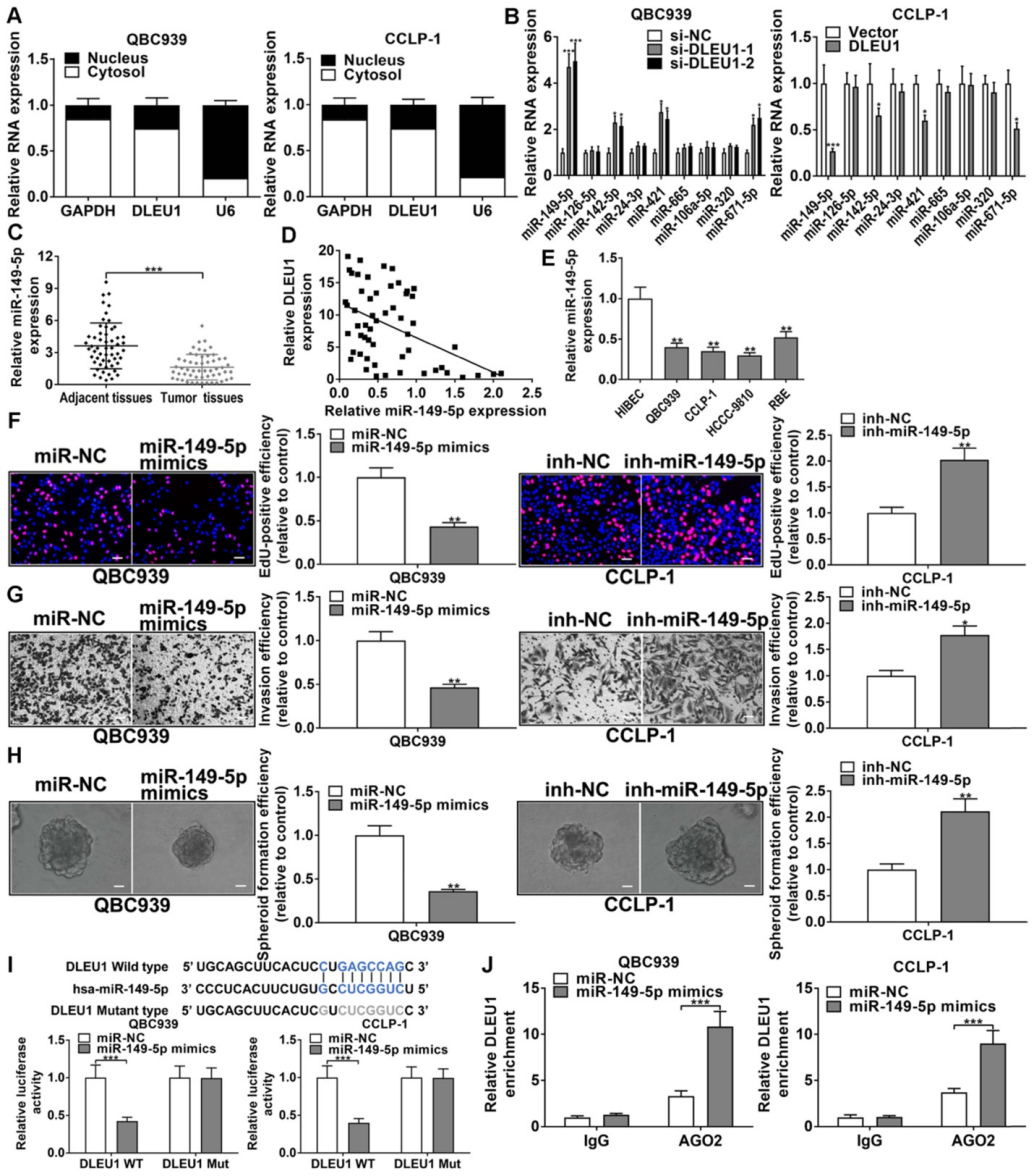
** DLEU1 competitively bound miR-149-5p in CCA. (A)** DLEU1 was affirmed to be principally expressed in cytoplasm. **(B)** MiRNAs levels following DLEU1 interference (two-way ANOVA). **(C)** MiR-149-5p level was downregulated in CCA tissues (*t*-test). **(D)** MiR-149-5p level was inversely linked to DLEU1 level (linear regression). **(E)** MiR-149-5p level in CCA cells and biliary epithelial cells (one-way ANOVA). **(F-H)** The function of miR-149-5p on growth, invasion, and stemness maintenance were revealed by EdU, transwell and spheroid formation assays (*t*-test). **(I)** MiR-149-5p mimics restrained luciferase activity of DLEU1 wild-type plasmid rather than mutant-type plasmid (*t*-test). **(J)** DLEU1 level was higher in AGO2 groups with miR-149-5p mimics (*t*-test). ^*^p < 0.05, ^**^p < 0.01, ^***^p < 0.001.

DLEU1 was affirmed to be principally expressed in cytoplasm (Figure 5A). By StarBase v3.0 (http://starbase.sysu.edu.cn/) prediction, DLEU1 as a ceRNA could bind some miRNAs, and miR-149-5p was determined for further study on the basis of qRT-PCR data (Figure 5B). The efficiencies of miR-149-5p inhibitor and mimics in QBC939 and CCLP-1 were satisfactory (Supplementary Figure 3B). Furthermore, miR-149-5p level was reduced in CCA tissues (Figure 5C), and decreased miR-149-5p was inversely linked to DLEU1 level (r = -0.4610, p < 0.001; Figure 5D). MiR-149-5p was also reduced in CCA cells (Figure 5E). To detect miR-149-5p function on the malignant biological behavior of CCA cells, we performed the CCK-8, EdU, colony formation, wound healing, transwell, spheroid formation, and western blot assays. The results confirmed that upregulated miR-149-5p restrained proliferation, invasion, and stemness maintenance of CCA cells, whereas knocking down miR-149-5p promoted cellular proliferation, invasion, and stemness maintenance (Figures 5F-H, Supplementary Figure 4). Furthermore, the binding site of miR-149-5p and DLEU1 were detected by StarBase v3.0. The luciferase reporter assays showed that miR-149-5p mimics restrained luciferase activity of DLEU1 wild-type plasmid rather than mutant-type plasmid (Figure 5I). The result confirmed that DLEU1 bound directly to miR-149-5p through this binding site. RIP assays showed that DLEU1 level was higher in AGO2 groups with miR-149-5p mimics (Figure 5J), further suggesting the targeted binding of DLEU1 to miR-149-5p.

### MiR-149-5p targeted YAP1 in CCA

**Figure 6:**
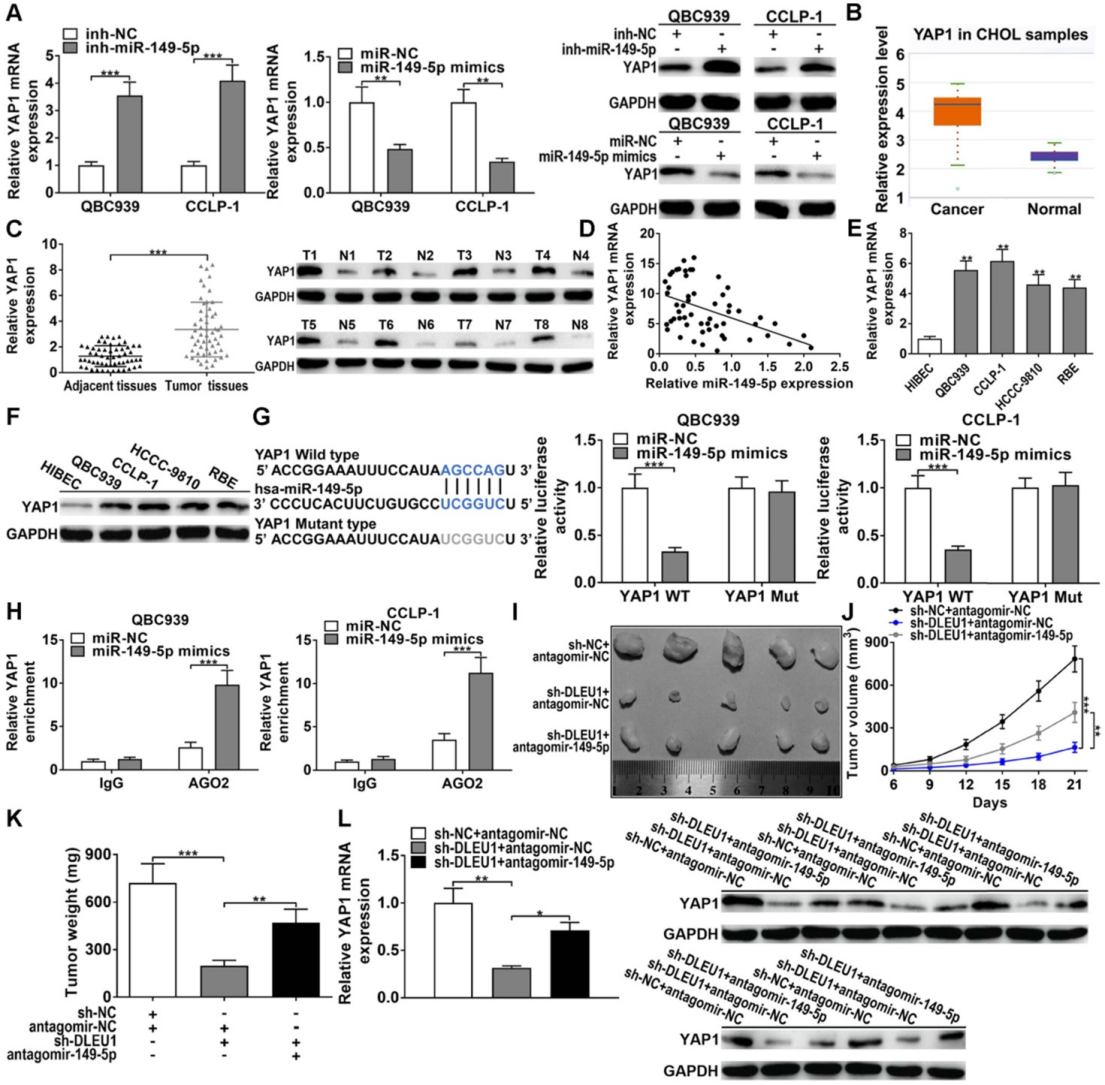
** MiR-149-5p targeted YAP1 in CCA. (A)** MiR-149-5p downexpression promoted YAP1 expression and miR-149-5p mimics inhibited YAP1 expression (*t*-test). **(B)** Detection of YAP1 level in CCA samples of TCGA database (*t*-test). **(C)** YAP1 level was upregulated in CCA tissues (*t*-test). **(D)** YAP1 level was inversely linked to miR-149-5p level (linear regression). **(E-F)** YAP1 level in CCA cells and biliary epithelial cells (one-way ANOVA). **(G)** MiR-149-5p mimics restrained luciferase activity of YAP1 wild-type plasmid rather than mutant-type plasmid (*t*-test). **(H)** YAP1 level was higher in AGO2 groups with miR-149-5p mimics (*t*-test). **(I)** QBC939 cells cotransfected with sh-DLEU1 and antagomir-149-5p were subcutaneously inoculated into mice, and **(J)** tumor volumes (two-way ANOVA) and **(K)** tumor weights (*t*-test) were depressed by knocking down DLEU1, but decreased miR-149-5p could partly rescue the inhibitory effect. **(L)** YAP1 mRNA and protein in xenograft tumors (*t*-test). ^*^p < 0.05, ^**^p < 0.01, ^***^p < 0.001.

StarBase forecasted that miR-149-5p could sponge YAP1 by the binding site, which overlapped the site between DLEU1 and miR-149-5p. The silencing efficiency of si-YAP1 and amplification efficiency of pcDNA3.1-YAP1 in QBC939 and CCLP-1 were shown in Supplementary Figure 5. As displayed in Figures 6A, decreased miR-149-5p promoted YAP1 expression, whereas upregulated miR-149-5p inhibited the expression of YAP1 in QBC939 and CCLP-1 cells. YAP1 level was enhanced in CCA via TCGA (p < 0.001; Figure 6B). Via qRT-PCR and western blot analysis, YAP1 level was indeed enhanced in CCA tissues (Figures 6C). Besides, YAP1 level was inversely linked to miR-149-5p level (r = -0.4762, p < 0.001; Figure 6D). YAP1 was enhanced in CCA cells as well (Figures 6E, F). By loss- and gain-of-function experiments, we confirmed that YAP1 knockdown restrained the proliferation, invasion, and stemness maintenance of CCA cells, while overexpressing YAP1 promoted these malignant phenotypes (Supplementary Figure 6). Afterwards, luciferase assay testified that miR-149-5p mimics depressed luciferase activity of YAP1 wild-type plasmid rather than mutant-type plasmid (Figure 6G). AGO2 RIP experiments further testified that miR-149-5p markedly enriched YAP1 mRNA (Figure 6H). Above data illustrated that miR-149-5p could sponge YAP1 to restrain YAP1 level.

For *in vivo* experiments, DLEU1 knockdown repressed tumor volumes and tumor weights, but cotransfected antagomir-149-5p could partly save the inhibitory function of silencing DLEU1* in vivo* (Figures 6I-K). Furthermore, YAP1 level in tumor tissues was measured. Silencing DLEU1 depressed YAP1 level both at mRNA and protein levels, while downregulated miR-149-5p partly saved the inhibitory effect generated via silencing DLEU1 (Figure 6L). Above data illustrated that DLEU1/miR-149-5p/YAP1 contributed to CCA tumorigenesis* in vivo*.

### YY1-induced DLEU1 boosted CCA progression via competitively binding miR-149-5p to elevate YAP1

**Figure 7:**
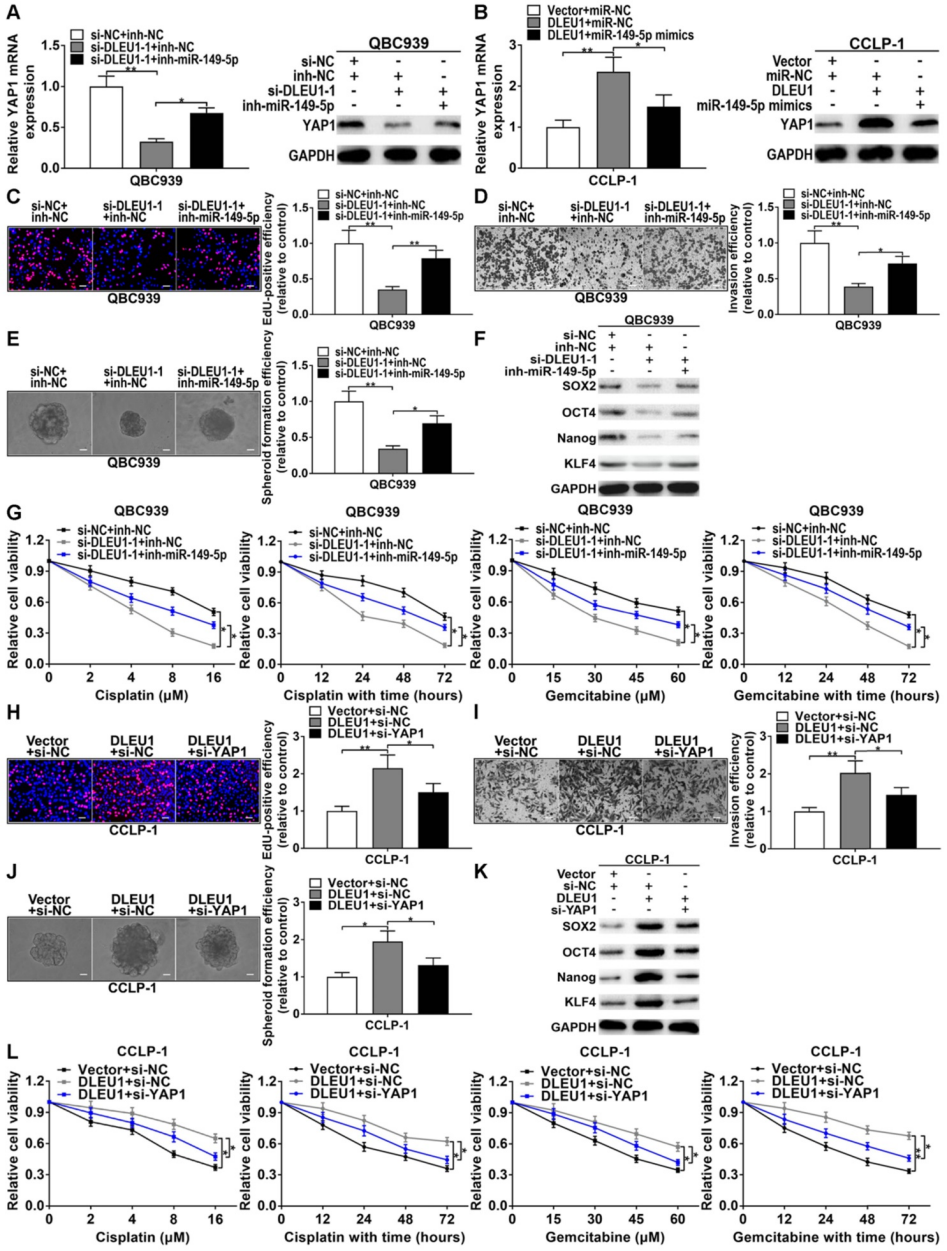
** DLEU1 boosted CCA development via miR-149-5p/YAP1. (A)** YAP1 inhibition generated via silencing DLEU1 was rescued via knocking down miR-149-5p (*t*-test). **(B)** YAP1 overexpression caused by DLEU1 upregulation was rescued via upregulating miR-149-5p (*t*-test). **(C-G)** Suppression of proliferation, invasion, stemness maintenance and chemo-resistance generated via silencing DLEU1 was rescued via miR-149-5p inhibitor in QBC939 (*t*-test and two-way ANOVA). **(H-L)** Decreased YAP1 saved the facilitation of proliferation, invasion, stemness maintenance and chemo-resistance caused via DLEU1 overexpression (*t*-test and two-way ANOVA).^ *^p < 0.05, ^**^p < 0.01.

Silencing YY1 promoted miR-149-5p expression, whereas upregulated YY1 inhibited miR-149-5p expression (Supplementary Figure 7A). And silencing YY1 inhibited YAP1 expression, whereas upregulated YY1 promoted YAP1 expression (Supplementary Figure 7B, C). The correlation between DLEU1 and YAP1 was positively correlated (r = 0.5433, p < 0.001; Supplementary Figure 7D). Silencing DLEU1 depressed YAP1 level, while simultaneously silencing miR-149-5p could partly reverse the function of DLEU1 both at mRNA and protein levels (Figure 7A). Likewise, DLEU1 overexpression promoted YAP1 expression, but upregulated miR-149-5p could partly save DLEU1-induced promotion both at mRNA and protein levels (Figure 7B). Furthermore, silencing DLEU1 depressed QBC939 proliferation, but miR-149-5p inhibitor could partly save the effect of DLEU1 (Figure 7C). Silencing miR-149-5p also partly saved the invasive suppression generated via silencing DLEU1 (Figure 7D). Moreover, decreased DLEU1 depressed spheroid formation, stem cell marker level, and sensitized QBC939 to cisplatin and gemcitabine, but miR-149-5p inhibitor partly rescued the function of silencing DLEU1 (Figures 7E-G). In addition, decreased YAP1 partly rescued the promotion of proliferation and invasion generated via DLEU1 upregulation (Figures 7H, I). Likewise, decreased YAP1 rescued the promotion of stemness maintenance and chemo-resistance induced via DLEU1 upregulation (Figures 7J-L). Furthermore, cancer-promoting effect caused via silencing miR-149-5p was partly saved by YAP1 downregulation (Supplementary Figure 7E-I). Above data illustrated that YY1-mediated DLEU1 upregulated YAP1 via sponging miR-149-5p, thereby boosting CCA development.

### YAP1/TEAD2 promoted stemness maintenance by transcriptionally upregulating SOX2

**Figure 8:**
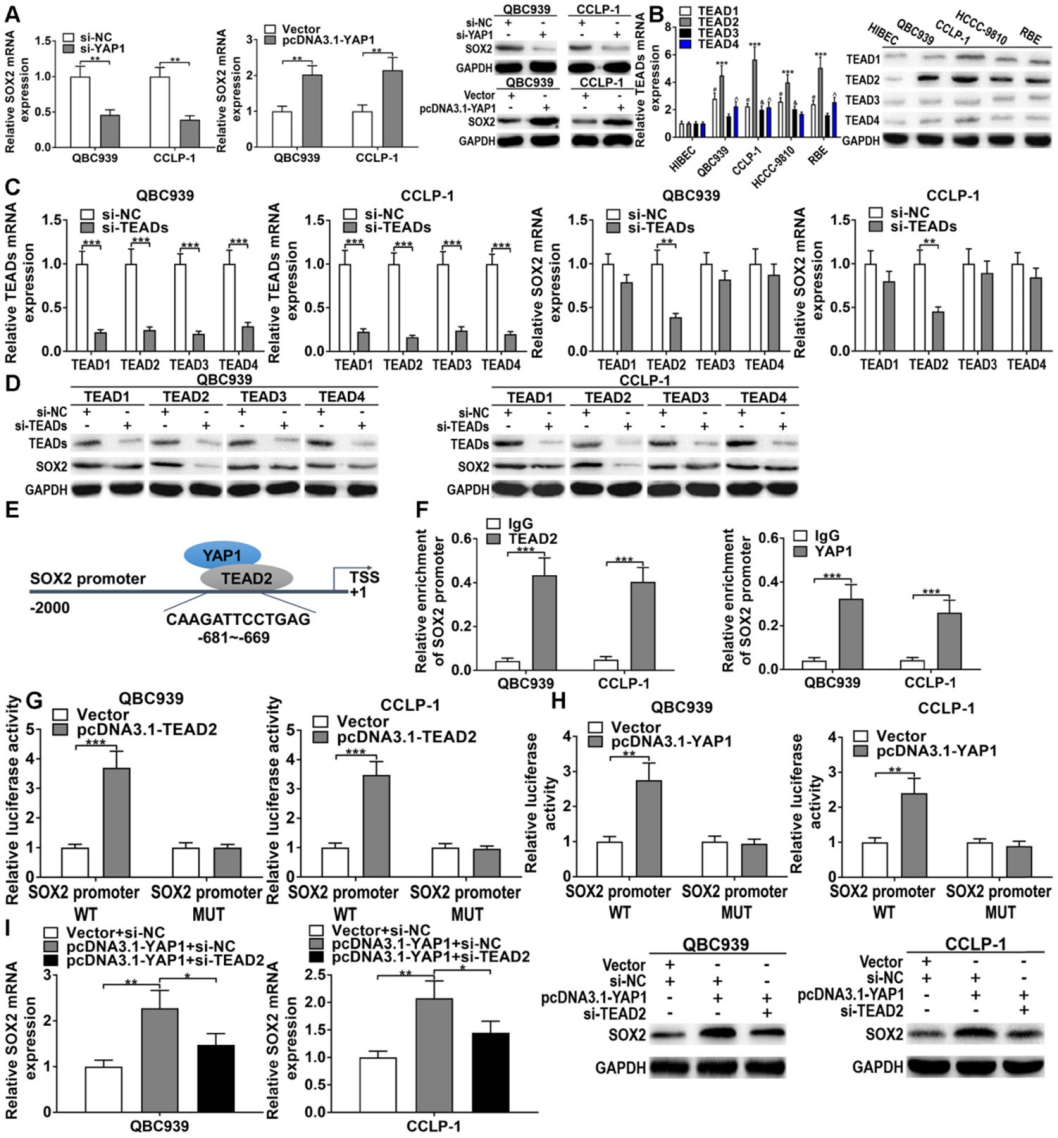
** YAP1/TEAD2 promoted stemness maintenance by upregulating SOX2. (A)** Knocking down YAP1 inhibited SOX2 expression and upregulated YAP1 promoted SOX2 expression testified by qRT-PCR and western blot (*t*-test). **(B)** The TEADs level in CCA cells and biliary epithelial cells (two-way ANOVA). **(C-D)** The knockdown efficiencies of si-TEADs and the effect of si-TEADs on SOX2 expression confirmed by qRT-PCR and western blot (two-way ANOVA). **(E)** The binding site of TEAD2 on SOX2 promoter was forecasted via JASPAR. **(F)** ChIP assays confirmed that YAP1/TEAD2 transcriptionally activated SOX2 in QBC939 and CCLP-1 cells (*t*-test). **(G-H)** The luciferase activity of SOX2 promoter wild type was promoted by TEAD2 and YAP1 plasmids (*t*-test). **(I)** SOX2 overexpression caused by YAP1 amplification was saved by knocking down TEAD2 (*t*-test). ^*^p < 0.05, ^**^p < 0.01, ^***^p < 0.001.

Oncogene YAP1 plays an important role in maintaining tumor stemness. Thus we examined the effect of YAP1 on stem cell marker in CCA cells. As shown in Figure 8A, knocking down YAP1 inhibited SOX2 expression and YAP1 overexpression promoted SOX2 level in CCA cells. As a transcription coactivator, YAP1 does not bind directly to the promoter regions of target genes. Therefore, it usually binds to DNA-binding transcription factor TEAD to regulate target gene expression. By JASPAR analysis, we found existence of the binding sites of TEAD in SOX2 promoter regions. Expressions of TEAD four subtypes were detected in CCA cells, and TEAD2 expression was significantly increased compared with other subtypes (Figure 8B). Knocking down TEAD four subtypes significantly restrained the expressions of TEADs, however just si-TEAD2 markedly suppressed SOX2 expression both at mRNA and protein levels (Figures 8C, D). The binding site of TEAD2 to SOX2 promoters was displayed in Figure 8E. ChIP affirmed that TEAD2 antibody obviously recruited the binding fragments of SOX2 promoters, and the promoter regions of SOX2 were also enriched by YAP1 antibody (Figure 8F). The amplification efficiency of pcDNA3.1-TEAD2 was shown in Supplementary Figure 8. Luciferase reporter assays further confirmed that overexpressed TEAD2 promoted the luciferase activity of the wild type of SOX2 promoter, whereas overexpressed TEAD2 had no impact on mutant type (Figure 8G). Consistent with these results, overexpressed YAP1 only boosted the luciferase activity of the wild type of SOX2 promoter (Figure 8H). In addition, YAP1 overexpression promoted the expression of SOX2, while silencing TEAD2 could partly reverse the promoting function of pcDNA3.1-YAP1 both at mRNA and protein levels (Figure 8I). These findings indicated that oncogene YAP1 promoted stemness maintenance by binding to transcription factor TEAD2 to transcriptionally upregulating SOX2.

## Discussion

Emerging studies have revealed that lncRNAs exhibited a critical role in CCA progression 23,24. For example, ZEB1-AS1 represented awful survival and facilitated tumor growth by modulated miR-133b in CCA 23; MEG3 was downexpressed and dramatically related to clinical stages and awful prognosis in CCA 24. DLEU1 as a carcinogen has been shown to promote cancer progression. For instance, DLEU1 expression was upregulated and promoted tumor proliferation and invasion by sponging miR‐133a in hepatocellular carcinoma 11; DLEU1 predicted awful survival of gastric cancer and contributed to tumor growth 14. The present research first discussed the regulatory function of DLEU1 in CCA.

We analyzed the differentially expressed lncRNAs in the gene expression profile of CCA from TCGA database, and identified the significantly upregulated DLEU1 in CCA. Via qRT-PCR verification, DLEU1 was enhanced in CCA tissues and linked to poor clinicopathological parameters and survival. Moreover, DLEU1 was an independent risk indicator for CCA prognosis with satisfactory sensitivity and specificity. Accordingly, DLEU1 possessed enormous value to assess CCA prognosis. In this research, DLEU1 boosted tumor growth* in vivo* and *in vitro*, and also facilitated CCA cell invasion and EMT. Furthermore, DLEU1 boosted tumor stemness and chemo-resistance of cisplatin and gemcitabine *in vivo* and *in vitro*. The data illustrated that DLEU1 contributed to CCA growth, metastasis and chemo-resistance, and possessed tremendous potential to conquer CCA.

The functions of YAP1 in stemness maintenance, chemo-resistance, proliferation and invasion are widely investigated in tumors including CCA 25,26. For example, YAP1 together with TEADs transcriptionally activated pro-angiogenic MFAP5 to boost tube formation of human microvascular endothelial cells in CCA 27. By JASPAR and StarBase prediction, we found that DLEU1 could be induced by transcription factor YY1, and DLEU1 and YAP1 had repeated miR-149-5p binding sequences. Moreover, YAP1 was overexpression and miR-149-5p was downexpression in CCA by TCGA, qRT-PCR and western blot. The gain- and loss-of-function experiments confirmed that YAP1 boosted CCA cell growth, invasion and stemness maintenance, whereas miR-149-5p depressed the malignant biological behavior of CCA cells. Furthermore, we confirmed that YY1-induced DLEU1 as a ceRNA boosted YAP1 expression via competitively binding to miR-149-5p. YAP1 as a transcription coactivator has been confirmed to activate transcriptional targets by binding to TEADs in CCA 27. By JASPAR analysis, we found existence of the binding sites of TEAD in SOX2 promoter regions. And we further confirmed that YAP1 promoted stemness maintenance by binding to transcription factor TEAD2 to transcriptionally upregulating SOX2 in CCA.

To sum up, DLEU1 was elevated and linked to poor prognosis in CCA. Furthermore, DLEU1 was activated by YY1. YY1-mediated DLEU1 boosted CCA malignant development via competitively binding to miR-149-5p with YAP1. Furthermore, YAP1/TEAD2 promoted stemness maintenance by transcriptionally upregulating SOX2. Taken together, YY1/DLEU1/miR-149-5p/YAP1 axis exerts crucial cancer-promoting function in CCA progression, and DLEU1 has potentiality as a biomarker or therapeutic target.

## Supplementary Material

Supplementary figures and table.

## Figures and Tables

**Table 1:** Correlation between DLEU1 expression and clinicopathological characteristics of CCA patients

Clinicopathological parameters	Total cases (n = 55)	DLEU1 expression		p-value
		Low (n = 26)	High (n = 29)	
**Age (years)**				
< 60	15	9	6	0.364
≥ 60	40	17	23	
**Gender**				
Male	22	9	13	0.583
Female	33	17	16	
**Tumor location**				
Intrahepatic	24	13	11	0.422
Extrahepatic	31	13	18	
**Histological type**				
Adenocarcinoma	50	25	25	0.355
Non-adenocarcinoma	5	1	4	
**Differentiation grade**				
Well/moderate	26	15	11	0.181
Poor/undifferentiated	29	11	18	
**TNM stage**				
I-II	19	14	5	0.006^**^
III-IV	36	12	24	
**Lymph node invasion**				
Yes	34	12	22	0.030^*^
No	21	14	7	
**HBV infection**				
Positive	14	6	8	0.764
Negative	41	20	21	
**Serum CA19-9 level**				
> 37 U/ml	37	15	22	0.249
≤ 37 U/ml	18	11	7	
**Serum CEA level**				
> 5 ng/ml	35	14	21	0.173
≤ 5 ng/ml	20	12	8	

*Note.* ^*^p < 0.05, ^**^p < 0.01. Chi-square test. DLEU1, deleted in lymphocytic leukemia 1; CCA, cholangiocarcinoma.

**Table 2:** Univariate and multivariate analyses for overall survival of CCA patients

Variables	Univariate analysis			Multivariate analysis		
	HR	95% CI	P-value	HR	95% CI	P-value
**Age (years)**	1.383	0.757-2.525	0.292			
≥ 60 *vs*. < 60						
**Gender**	0.836	0.470-1.485	0.540			
Male *vs*. Female						
**Tumor location**	1.251	0.709-2.209	0.439			
Extrahepatic *vs*. Intrahepatic						
**Histological type**	0.781	0.279-2.187	0.638			
Adenocarcinoma* vs*. Non-adenocarcinoma						
**Differentiation grade**	1.546	0.875-2.732	0.134			
Poor/undifferentiated *vs*. Well/moderate						
**HBV infection**	1.290	0.731-2.278	0.380			
Positive* vs*. Negative						
**Serum CA19-9 level**	1.418	0.802-2.507	0.229			
> 37 U/ml *vs*. ≤ 37 U/ml						
**Serum CEA level**	1.670	0.914-3.050	0.095			
> 5 ng/ml* vs*. ≤ 5 ng/ml						
**TNM stage**	2.052	1.120-3.761	0.020^*^	2.327	1.268-4.272	0.006^**^
III-IV *vs*. I-II						
**Lymph node invasion**	1.921	1.039-3.554	0.037^*^	1.793	0.968-3.321	0.063
Yes *vs*. No						
**DLEU1 expression**	2.136	1.166-3.915	0.014^*^	2.586	1.429-4.682	0.002^**^
Low *vs*. High						

*Note.* ^*^p < 0.05, ^**^p < 0.01. Cox regression analysis. CCA, cholangiocarcinoma; HR, hazard ratio; CI, confidence interval; DLEU1, deleted in lymphocytic leukemia 1.

## Abbreviations

CCA: cholangiocarcinoma
DLEU1: deleted in lymphocytic leukemia 1
YY1: Yin Yang 1
YAP1: Yes-associated protein 1
TEAD: transcriptional enhanced associated domain
SOX2: SRY-box 2
ceRNA: competitive endogenous RNA
CSCs: cancer stem cells
OCT4: octamer-binding protein 4
KLF4: Kruppel-like factor 4
FBS: fetal bovine saline
GAPDH: glyceraldehyde 3-phosphate dehydrogenase
PVDF: polyvinylidene fluoride
EdU: 5-ethynyl-2'-deoxyuridine
PBS: phosphate-buffered saline
ChIP: chromatin immunoprecipitation
RIP: RNA immunoprecipitation
ROC: receiver operating characteristic curve
AUC: value of area under curve
TFBS: transcription factor binding site
