# Supplementary material for: YY1-induced DLEU1/miR-149-5p Promotes Malignant Biological Behavior of Cholangiocarcinoma through Upregulating YAP1/TEAD2/SOX2
Source: Int J Biol Sci. 2022 Jul 4;18(11):4301–15. doi: 10.7150/ijbs.66224 (PMC9295058; doi:10.7150/ijbs.66224)
Supplement: Supplementary file 1 — Supplementary figures and table. [file ijbsv18p4301s1.pdf]

Supporting Figure S1

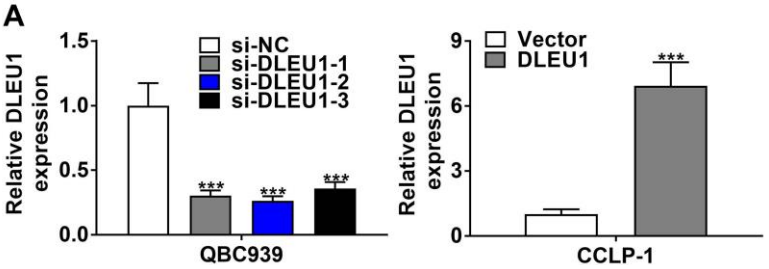

Figure S1. The efficiencies of DLEU1 siRNA and DLEU1 plasmid in CCA cells (*t*-test). ***p < 0.001.

Supporting Figure S2

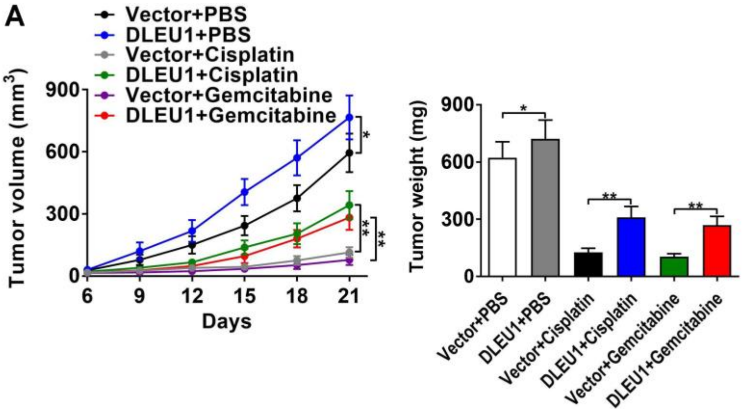

Figure S2. The tumor growth curve (two-way ANOVA) and tumor weight (*t*-test) of each groups were analysed. *p < 0.05, **p < 0.01.

Supporting Figure S3

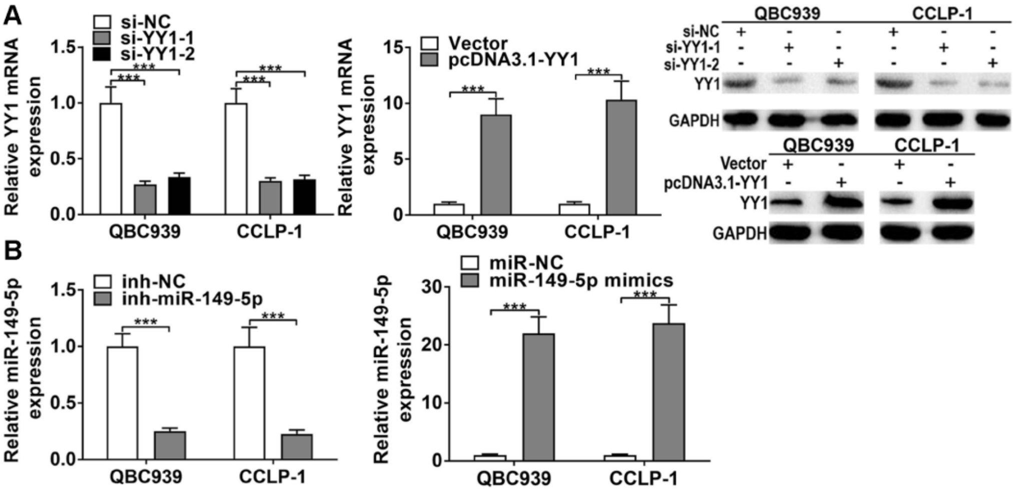

Figure S3. The knockdown efficiency and amplification efficiency. (A) The efficiencies of YY1 siRNA and YY1 plasmid in CCA cells (*t*-test). (B) The knockdown and amplification efficiencies of miR-149-5p in CCA cells (*t*-test). ***p < 0.001.

Supporting Figure S4

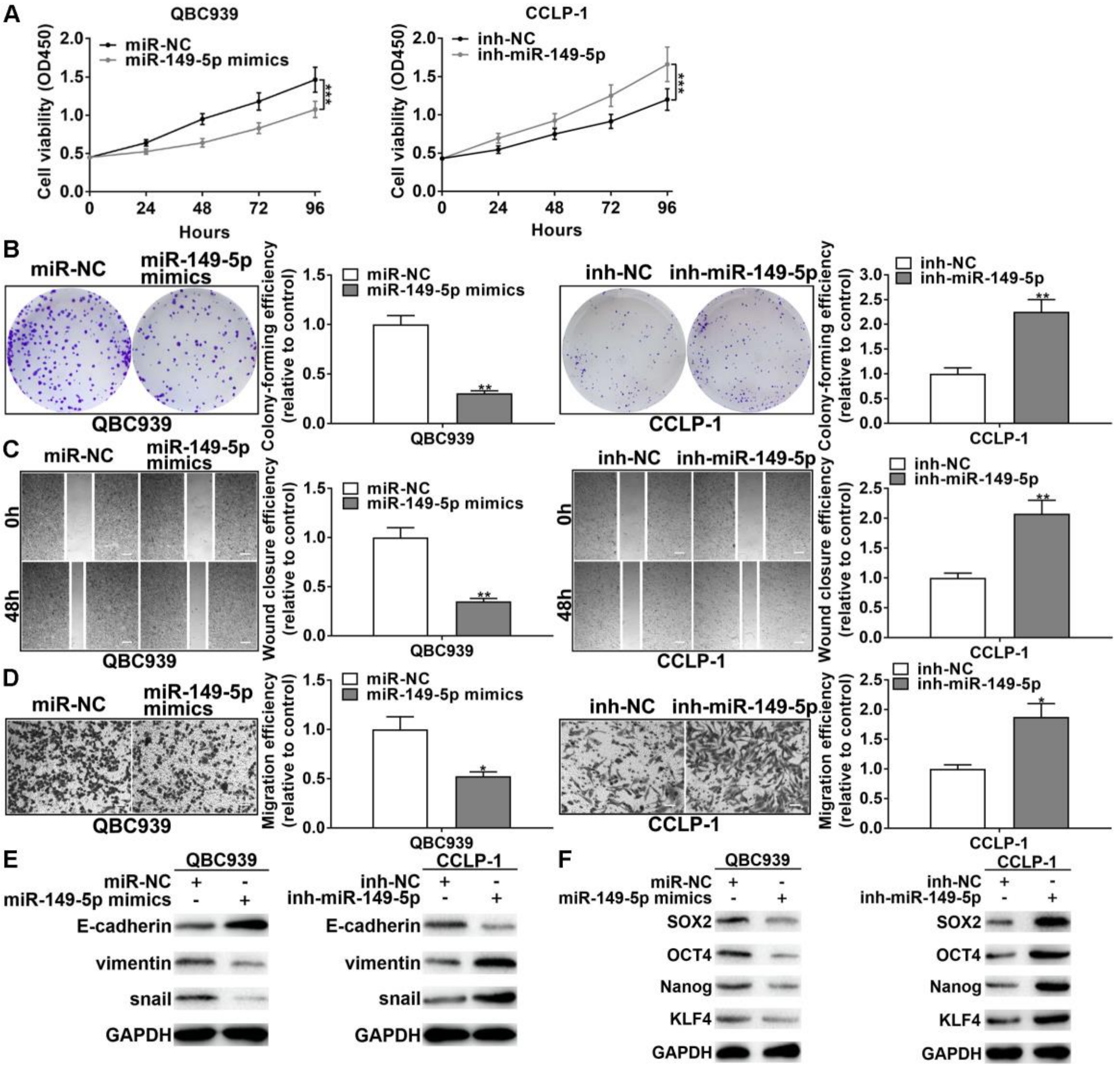

Figure S4. MiR-149-5p inhibited the proliferation, invasion and stemness maintenance of CCA cells. (A-B) The effects of miR-149-5p on CCA proliferation were detected by CCK-8 (two-way ANOVA) and colony formation assays (*t*-test). (C-D) The gain- and loss-of-function experiments about cell migration in QBC939 and CCLP-1 cells (*t*-test). (E) EMT-related proteins including E-cadherin, snail and vimentin were measured via western blot (two-way ANOVA). (F) The functions of miR-149-5p on stem markers (SOX2, OCT4, Nanog, KLF4) were detected by western blot (two-way ANOVA). The data were expressed as mean ± SD of three independent experiments. $*p < 0.05$, $**p < 0.01$, $***p < 0.001$.

Supporting Figure S5

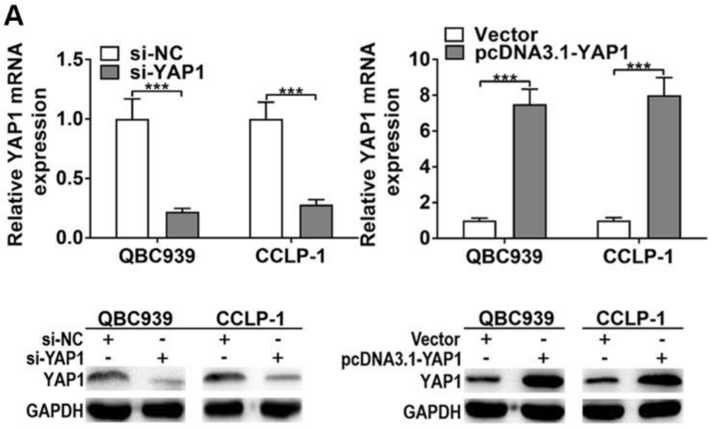

Figure S5. The knockdown efficiency of si-YAP1 and amplification efficiency of pcDNA3.1-YAP1 in QBC939 and CCLP-1 cells (*t*-test). ***$p < 0.001$.

Supporting Figure S6

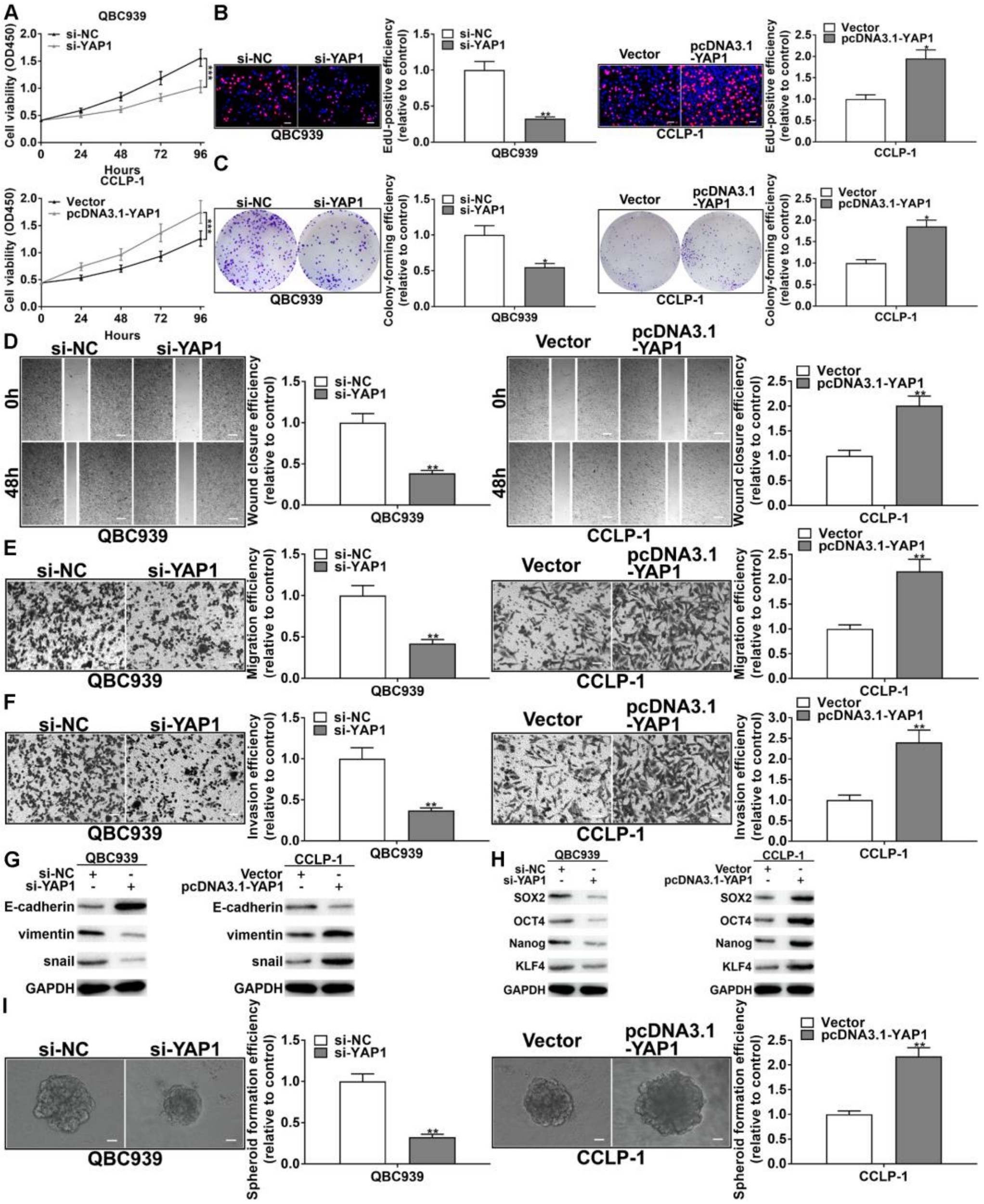

Figure S6. YAP1 promoted the proliferation, invasion and stemness maintenance of CCA cells. (A-C) Knocking down YAP1 suppressed QBC939 cell proliferation, whereas overexpressing YAP1 promoted CCLP-1 cell proliferation (two-way ANOVA and *t*-test). (D-G) The effects of YAP1 on CCA migration, invasion and EMT process were detected by wound healing (*t*-test), transwell (*t*-test) and western blot assays (two-way ANOVA). (H-I) The gain- and loss-of-function experiments of YAP1 on stemness maintenance in QBC939 and CCLP-1 cells were performed with spheroid formation assay (*t*-test) and stem marker analysis (two-way ANOVA). The data were expressed as mean ± SD of three independent experiments. *p < 0.05, **p < 0.01, ***p < 0.001.

Supporting Figure S7

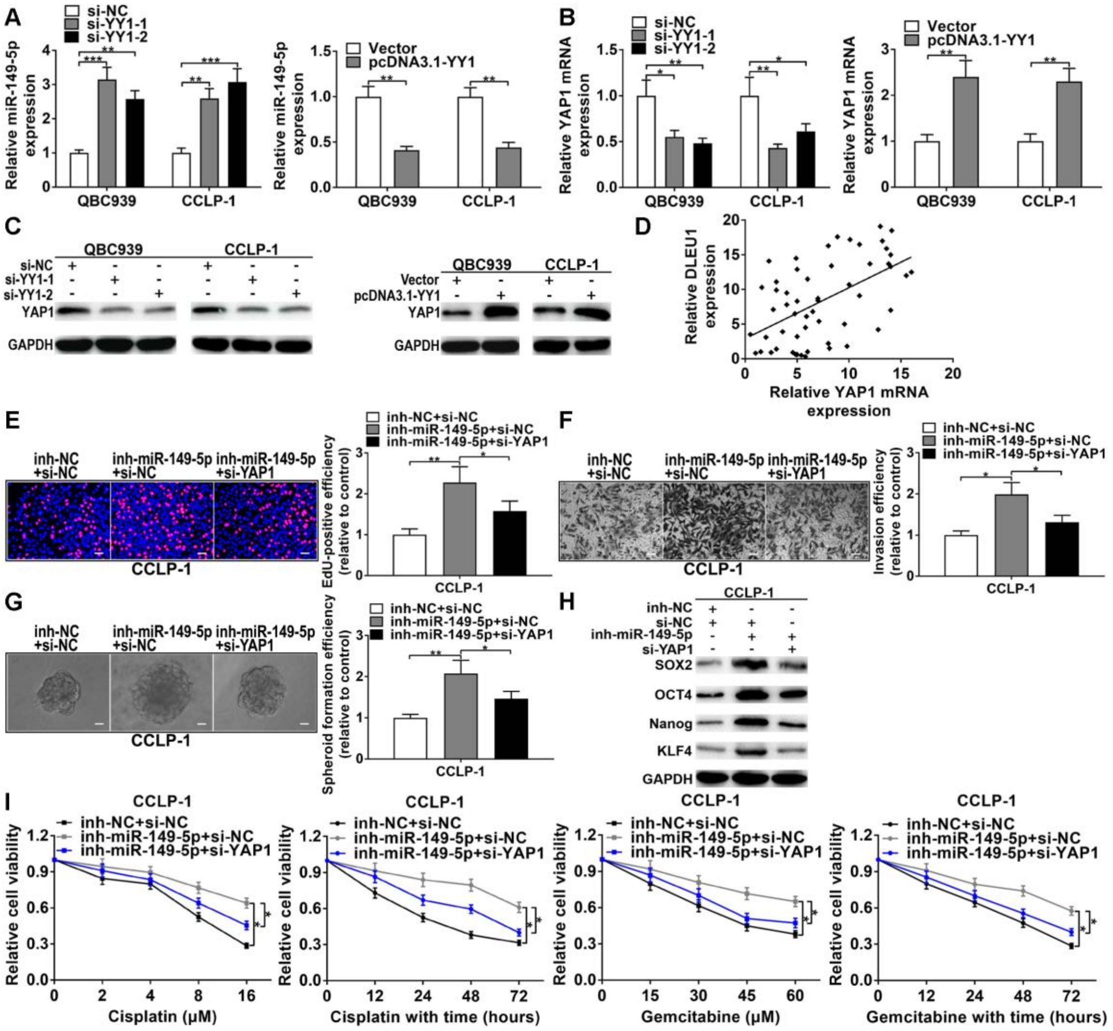

Figure S7. MiR-149-5p restrained CCA malignant progression by suppressing YAP1. (A) Silencing YY1 promoted miR-149-5p expression and upregulated YY1 inhibited miR-149-5p expression (*t*-test). (B-C) Silencing YY1 inhibited YAP1 expression and upregulated YY1 promoted YAP1 expression (*t*-test). (D) The correlation between DLEU1 and YAP1 was positively correlated (linear regression). (E-I) Rescue assays of EdU (*t*-test), transwell (*t*-test), spheroid formation (*t*-test), stem marker analysis (two-way ANOVA) and chemo-resistance (two-way ANOVA) confirmed that facilitation of proliferation, invasion, stemness maintenance and chemo-resistance induced by knocking down miR-149-5p was retrieved through silencing YAP1 in CCLP-1 cells, respectively. The data were expressed as mean ± SD of three independent experiments. *p < 0.05, **p < 0.01, ***p < 0.001.

Supporting Figure S8

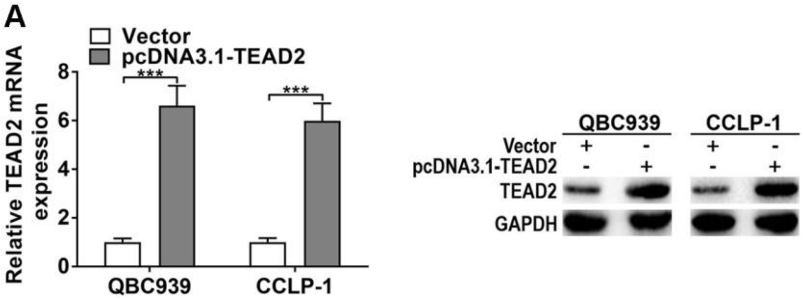

Figure S8. The amplification efficiency of pcDNA3.1-TEAD2 was analysed in CCA cells (*t*-test).

***$p < 0.001$.

Supporting Table S1

Table S1. Primer sequences for qRT-PCR and sequences for siRNA.

Nucleic acids	Sequences
DLEU1	Forward primer: 5’-TCAGAATGCCGACTCTATGCT-3’<br>Reverse primer: 5’-GGTGAGGACAGAGTTAAACGC-3’
GAPDH	Forward primer: 5’-GGGAGCCAAAAGGGTCAT-3’<br>Reverse primer: 5’-GAGTCCTTCCACGATACCAA-3’
YY1	Forward primer: 5’-AGCAGAAGCAGGTGCAGATCAA-3’<br>Reverse primer: 5’-CTGCCAGTTGTTTGGGATCT-3’
DLEU1 promoter E1 region	Forward primer: 5’-TTCAGGCTCTTACCGACTGC-3’<br>Reverse primer: 5’-GTCCGAGAGTATAGCGCCAC-3’
DLEU1 promoter E2 region	Forward primer: 5’-AAGAAAAGGTGGGGATCGCA-3’<br>Reverse primer: 5’-GGGGCTTCGAGCTAACGATT-3’
DLEU1 promoter E3 region	Forward primer: 5’-AGCCAATCAGGGGGTAACAAG-3’<br>Reverse primer: 5’-CACGCTTCTTTAGGACTGCC-3’
U6	Forward primer: 5’-GCTTCGGCAGCACATATACTAAAAT-3’<br>Reverse primer: 5’-CGCTTCACGAATTTGCGTGTCAT-3’
miR-149-5p	Forward primer: 5’-GGCTCTGGCTCCGTGTCTT-3’<br>Reverse primer: 5’-CAGTGCAGGGTCCGAGGTATT-3’
miR-126-5p	Forward primer: 5’-GGAATGTAAGGAAGTGTG-3’<br>Reverse primer: 5’-GAGCAGGCTGGAGAA-3’
miR-142-5p	Forward primer: 5’-GAAGATCTCCAGCCACCTGTTTCACA-3’<br>Reverse primer: 5’-CCGCTCGAGTAGTCCTTCACTTCATG-3’
miR-24-3p	Forward primer: 5’-GATCCTGGCTCAGTTCAGCAGGAACAGC-3’<br>Reverse primer: 5’-TCGAGCTGTTCCTGCTGAACTGAGCCAG-3’
miR-421	Forward primer: 5’-CTCACTCACATCAACAGACATTAATT-3’<br>Reverse primer: 5’-TATGGTTGTTCTGCTCTCTGTGTC-3’
miR-665	Forward primer: 5’-GGTCTACAAAGGGAAGC-3’<br>Reverse primer: 5’-TTTGGCACTAGCACATT-3’
miR-106a-5p	Forward primer: 5’-GATGCTCAAAAAGTGCTTACAGTGCA-3’<br>Reverse primer: 5’-TATGGTTGTTCTGCTCTCTGTCTC-3’
miR-320	Forward primer: 5’-AAATCAGGTTGCCGATCTTCA-3’<br>Reverse primer: 5’-GTGCAGGGTCCGAGGT-3’
miR-671-5p	Forward primer: 5’-GCCCGCAGGAAGCCCGGAGGGGC-3’<br>Reverse primer: 5’-GTGCAGGGTCCGAGGT-3’
YAP1	Forward primer: 5’-TAGCCCTGCGTAGCCAGTTA-3’<br>Reverse primer: 5’-TCATGCTTAGTCCACTGTCTGT-3’
SOX2	Forward primer: 5’-GCTACAGCATGATGCAGGACCA-3’<br>Reverse primer: 5’-TCTGCGAGCTGGTCATGGAGTT-3’
TEAD1	Forward primer: 5’-GCCTCCCAACATCCATAGCA-3’<br>Reverse primer: 5’-TCTGTCCACCAGCCGAGATT-3’
TEAD2	Forward primer: 5’-TGCCTTCTTCCTGGTCAAGTTC-3’<br>Reverse primer: 5’-GGCTCTCATACTGGCTGCTCA-3’

TEAD3	Forward primer: 5’-CATCGAGCAGAGCTTCCAG-3’<br>Reverse primer: 5’-CGTGCAATCAACTCATTTCG-3’
TEAD4	Forward primer: 5’-AGTCAGGCACTGGACAAGC-3’<br>Reverse primer: 5’-GCTGGAGACCTGCTTCCTG-3’
SOX2 promoter region	Forward primer: 5’-CTTACCAAGGCCTGCTGGTT-3’<br>Reverse primer: 5’-AGGTGCCAACACTCTCTCAC-3’
si-DLEU1-1	5’-GCAGUCUGUUCUGAACAUA-3’
si-DLEU1-2	5’-CAACGGAAUGUAUCAAUGA-3’
si-DLEU1-3	5’-GAGGGGUCGUCUUAUAGCUAC-3’
si-YY1-1	5’-GAACUCACCUCCUGAUUAU-3’
si-YY1-2	5’-UGCAGAUGCUUUCUCAUAGCAGAGU-3’
si-YAP1	5’-AGAUACUUCUUAAAUCACA-3’
si-TEAD1	5’-GGCCGAUUUGUAUACCGAA-3’
si-TEAD2	5’-CGAAGGAAAUCAAGGGAAA-3’
si-TEAD3	5’-UACCUUGCUCUCAAUCUGGAG-3’
si-TEAD4	5’-UUUCCUGCACACACGUCUCUU-3’

Note. qRT-PCR, quantitative real-time polymerase chain reaction; DLEU1, deleted in lymphocytic leukemia 1; GAPDH, glyceraldehyde 3-phosphate dehydrogenase; YY1, Yin Yang 1; YAP1, Yes-associated protein 1; SOX2, SRY-box 2; TEAD, transcriptional enhanced associated domain.
